# Seascapes of fear and competition shape regional seabird movement ecology

**DOI:** 10.1038/s42003-022-03151-z

**Published:** 2022-03-04

**Authors:** Nicolas Courbin, Lorien Pichegru, Mduduzi Seakamela, Azwianewi Makhado, Michael Meÿer, Pieter G. H. Kotze, Steven A. Mc Cue, Clara Péron, David Grémillet

**Affiliations:** 1 Centre d’Ecologie Fonctionnelle et Evolutive (CEFE), UMR 5175 CNRS – Université de Montpellier – Université Paul Valéry Montpellier 3 – EPHE – IRD, Montpellier, France; 2 Laboratoire d’Écologie Alpine (LECA), UMR 5553 Université Grenoble Alpes – Université Savoie Mont-Blanc – CNRS, Le Bourget-du-Lac, France; 3 Institute for Coastal and Marine Research, Nelson Mandela University, Gqeberha, South Africa; 4 Department of Forestry, Fisheries and the Environment, Branch Ocean and Coasts, Cape Town, South Africa; 5 Percy FitzPatrick Institute of African Ornithology, University of Cape Town, Rondebosch, South Africa; 6 Laboratoire de Biologie des Organismes et Ecosystèmes Aquatiques (UMR BOREA) MNHN, CNRS, IRD, SU, UCN, UA, Paris, France; 7 Centre d’Etudes Biologiques de Chizé (CEBC), UMR 7372 CNRS – La Rochelle Université, Villiers-en-Bois, France

**Keywords:** Behavioural ecology, Biooceanography

## Abstract

Fear effects of predators on prey distributions are seldom considered in marine environments, especially over large spatial scales and in conservation contexts. To fill these major gaps, we tested the Seascape of Fear Hypothesis in the Benguela marine ecosystem off South Africa. Using electronic tracking data, we showed that Cape gannets and their predator, the Cape fur seal, co-occurred in daytime and competed with fisheries within coastal areas. At night, gannets are particularly vulnerable to seals, and 28% of the birds returned to the safety of their breeding colony. The remaining 72% slept at the sea surface, but shifted to offshore areas with lower seal attendance, reducing predation risk by 25%. Overall, our integrative study demonstrates how fear and competition shape the seascape of threatened Cape gannets within a marine environment perturbed by climate change and overfishing. Such knowledge has strong implications for the design of marine protected areas.

## Abstract (web summary)

Fear effects of predators on prey distributions are seldom considered in marine environments, even though they have strong implications for the design of marine protected areas. This work demonstrates such ‘Seascape of Fear’ for Cape gannets off South Africa, as they adjusted their at-sea movements relative to those of predatory Cape fur seals.

## Introduction

Seascape ecology is on the rise. Decades after the advent of landscape ecology, its aquatic counterpart has taken a giant footstep^1^. Indeed, this fusion of geography and ecology benefited from geospatial revolution, with the rapid development of global data acquisition and mapping tools. Those allow assessing the effect of structural environmental conditions upon the biogeography of marine organisms. In this context, a major constraint is the fear effect, i.e., the spatial variation in prey perception of predation risk, termed the Landscape of Fear (LoF)^2^, and later extended to the Seascape of Fear (SoF)^3^. Fear effects modify prey behavior, induce diel migratory patterns^4^ and entail energetic costs, with cascading effects onto population dynamics and entire ecosystems^5,6^. The fearscape concept has gained substantial interest across the last decade, with renewed focus on temporal dynamics^4,5^. Surprisingly, such fear effects are not addressed by Pittman^1^ in their seminal work on seascape ecology.

We tested the SoF hypothesis at a regional scale in the ecologically perturbed Benguela upwelling ecosystem off South Africa. There, Cape gannets (*Morus capensis*) are at risk from predation by Cape fur seals (*Arctocephalus pusillus pusillus*), specifically when resting at the water surface during the night when they are most vulnerable to underwater predator attacks. Such predation events have been documented on land and at sea^7,8^ (Supplementary Data 1), but their impacts on Cape gannet at-sea behavior has never been tested, thereby blurring our understanding of non-consumptive effects at a regional scale. Moreover, gannets compete with seals and regional fisheries for the consumption of diminishing stocks of small pelagic fish (sardines *Sardinops sagax* and anchovies *Engraulis encrasicolus*), but are provisioned with fishery wastes dumped at sea by trawlers targeting hake (*Merlucius spp*.)^9^. In this context, we hypothesized that spatiotemporal variations in fear and competition contribute to shaping the spatial ecology of gannets in the Benguela upwelling ecosystem. Specifically, we predicted that (1) birds would avoid seals at all times, especially during the riskiest nighttime period. This was predicted to induce diel gannet spatial shifts at sea, towards safer areas at night. However, during the less risky daytime period, gannets have to balance predation risk by seals with their foraging needs. We predicted that (2) birds would compete with seals and fisheries while feeding at sea during daytime. Alternatively, we hypothesized that thermoregulatory constraints also shaped gannet at sea distributions. We took advantage of a large multi-year data set, which included electronic tracking of at-sea movements for seals and gannets in the Southern Benguela upwelling ecosystem, as well as the distribution of fishing activities and sea surface temperatures in this region. On the basis of this information, we assessed the at-sea spatial ecology of the different marine predators involved in the Benguela seascape.

## Results

**Fig. 1:**
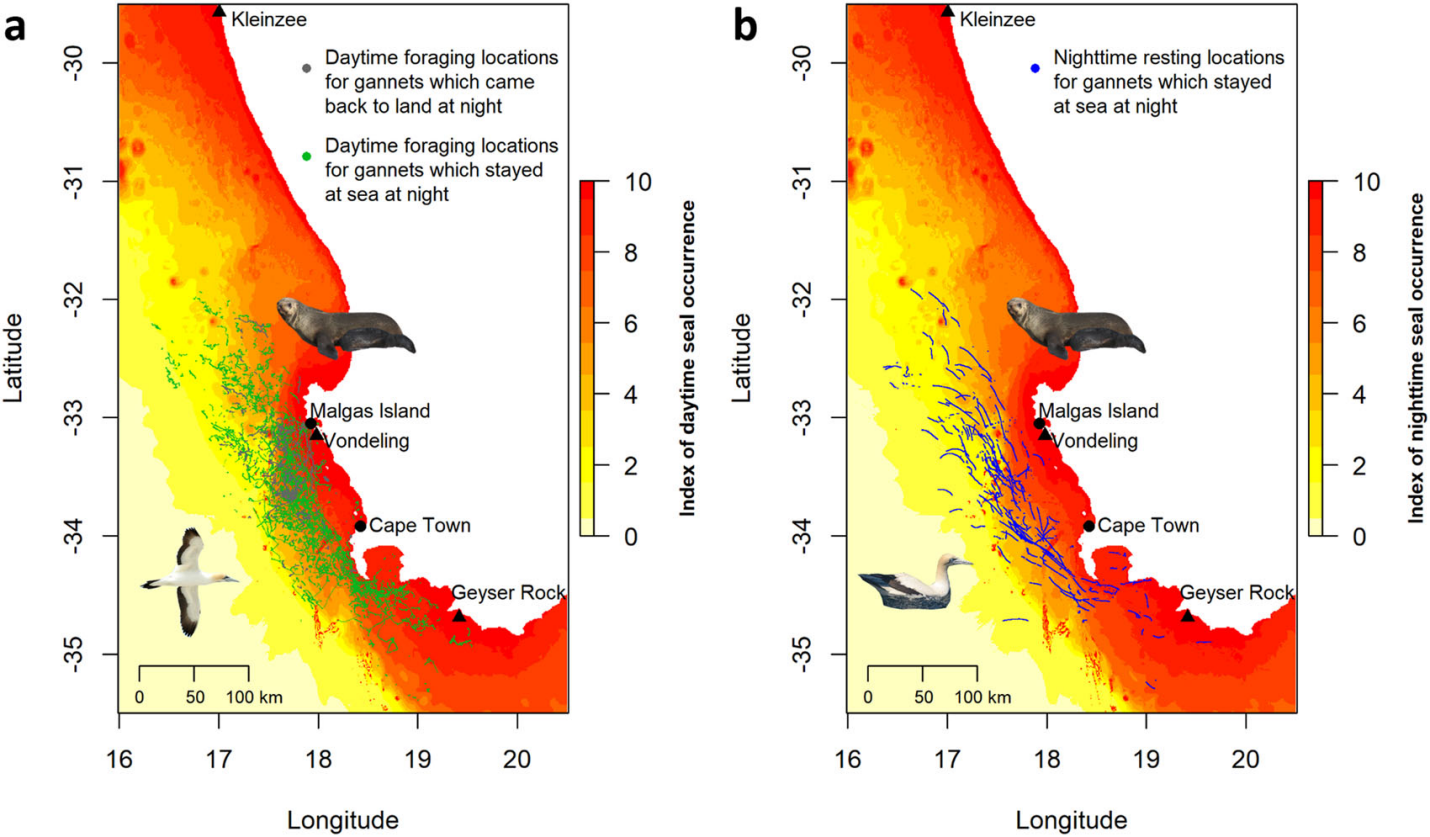
Seascape of fear induced by Cape fur seals for Cape gannets. **a** Daytime seascape of fear (yellow to red index of seal occurrence) and daytime foraging locations for gannets returning to the colony at night (gray dots) or resting at sea all night (green dots). **b** Nighttime seascape of fear (yellow to red index of seal occurrence) and gannet nighttime at-sea resting locations (blue dots). Seals (*n* = 25 individuals) were Argos-tracked from three colonies (black triangles) and gannets (*n* = 197 individuals) were GPS-tracked from Malgas Island (black dot).

**Fig. 2:**
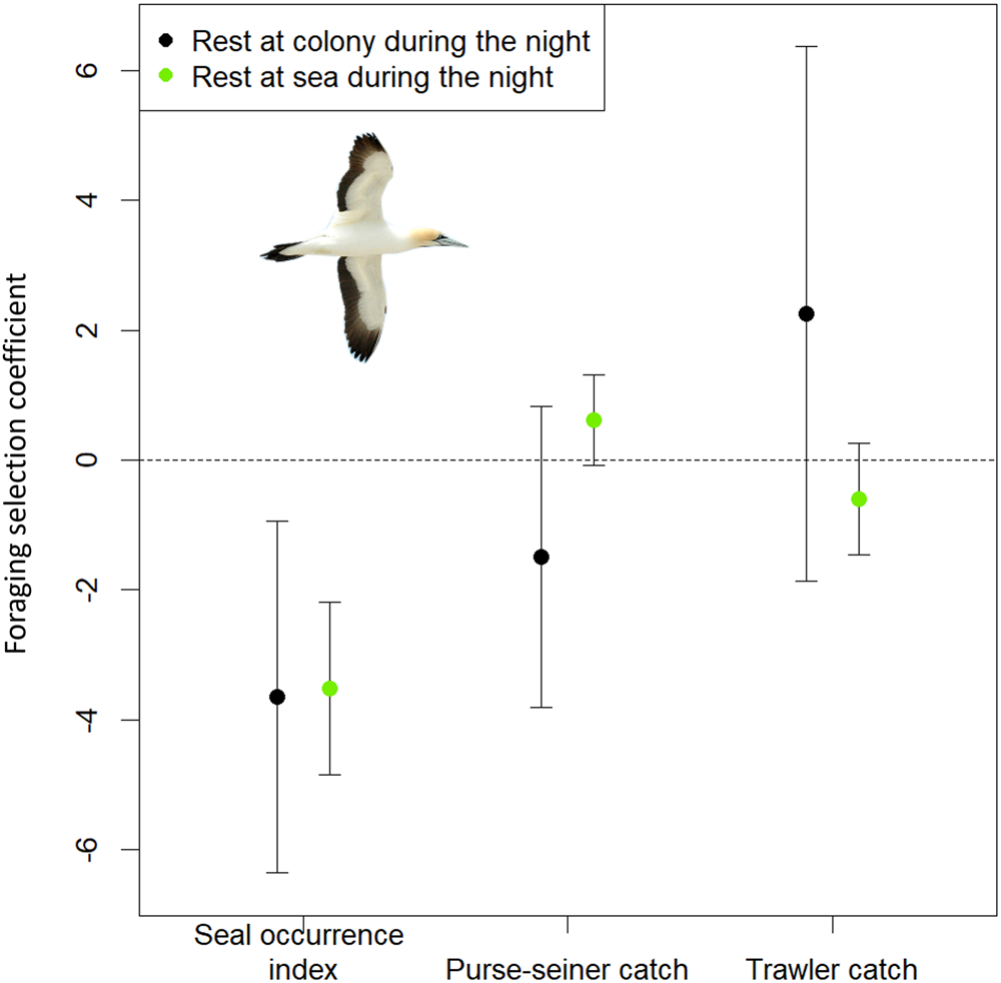
Daytime foraging habitat selection of Cape gannets. Habitat selection coefficients of foraging behavior during daytime, depending on nighttime resting tactics for 197 Cape gannets from Malgas Island during October–November between 2008 and 2015. Error bars represent the 95% confidence interval. Confidence interval including 0 means no selection.

**Fig. 3:**
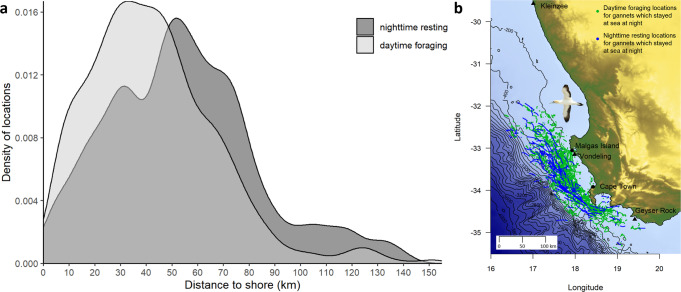
Diel spatial shift of 144 Cape gannets which stayed at sea at night during October–November between 2008 and 2015. **a** Distribution of gannet nighttime resting locations (dark gray) and daytime foraging locations (light gray) in relation to distance to shore. **b** At-sea spatial distribution of daytime foraging (green dot) and nighttime resting locations (blue dot) of gannets. Triangles show the three monitored seals colonies. Map generated with the *marmap*^41^ package for R.

**Fig. 4:**
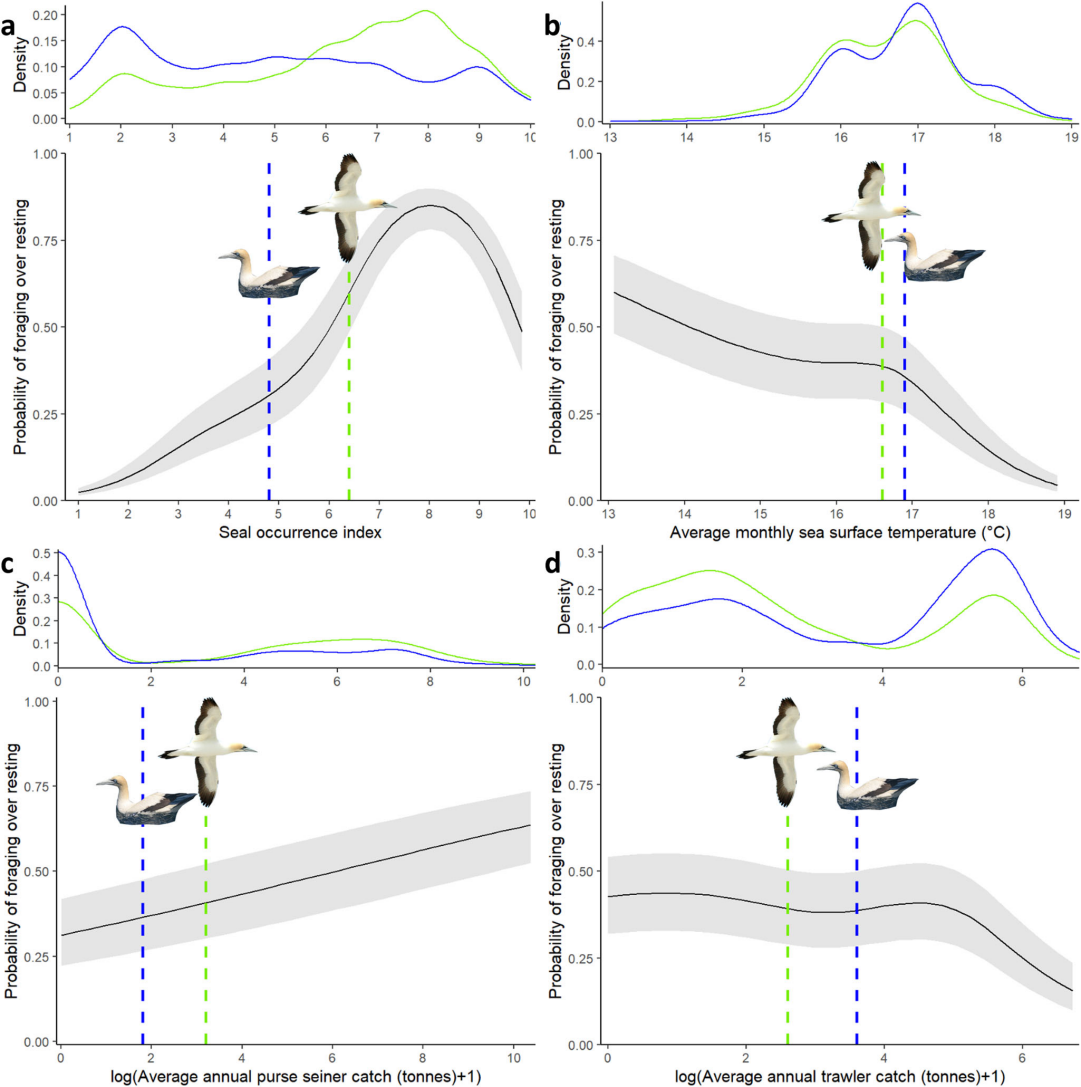
Probability of daytime foraging over nighttime resting for gannets in relation to environmental features (*n* = 142 birds). We predicted the effect of **a** seal occurrence, **b** sea surface temperature, **c** purse-seiner catches, and **d** trawler catches, with a mixed effect logistic regression and keeping all other covariates at their mean. Grey areas represent the 95% confidence interval. The x-axis is back-transformed (unscaled). Distribution and mean (dotted line) are shown for daytime foraging (green) and nighttime resting (blue) locations.

Testing prediction 1: Seals at sea were active night and day, with similar habitat selection patterns and a decrease in occurrence with increasing distance from the coast (Fig. 1, Supplementary Note 1, Supplementary Fig. 1, Supplementary Table 1). Gannets displayed two tactics when at sea: 1) Some (28%) came back to the colony at night after daytime foraging. 2) Most (72%) remained at sea in-between daytime foraging bouts, and spent 76% of nighttime resting at the sea surface (Fig. 1, Table 1, Supplementary Note 2). In agreement with our first prediction, all gannets, regardless of whether they remained at sea at night, or came back to the colony, selected foraging areas that decreased their risk of encountering seals during daytime (Fig. 2, Supplementary Note 3, Supplementary Table 2). Crucially, birds which rested at the sea surface overnight displayed an offshore spatial shift (13.1 km) between locations dedicated to daytime foraging and nighttime resting (Fig. 3, Table 1, Supplementary Note 4, Supplementary Table 3). This shift allowed resting gannets to decrease their risk of encountering seals by 25% at night, on average, whereas nighttime resting waters were on average only 0.3 °C warmer than daytime coastal foraging areas (Table 1, Supplementary Note 4). Indeed, the likelihood of nighttime resting over daytime foraging continuously increased with the decrease in seal occurrence, except in areas with high encounter risk, but gannets remained more likely to forage than rest in such areas (Fig. 4a, Supplementary Note 4, Supplementary Tables 4, 5). In contrast, gannets behaved equally with an increase in sea surface temperature, until high-temperature values where resting became more likely than foraging (Fig. 4b). Therefore, the large decrease in predation risk outperformed a small thermoregulatory benefit, on average. Nighttime periods spent at the sea surface may also allow adult gannets to digest a first food load, before catching another the next morning, to be brought back to their chick^10^.

**Table 1:** Summary statistics of habitat use for 197 Cape gannets.

Variable	Daytime foraging, spent night at colony *N* = 6890 locations, 53 birds		Daytime foraging, spent night at sea *N* = 47953 locations, 144 birds		Nighttime resting, spent night at sea *N* = 85142 locations, 142 birds	
	Mean (95% CI)	Min;max	Mean (95% CI)	Min;max	Mean (95% CI)	Min;max
Distance to shore (km)	41.9 (37.5;46.5)	0.0;95.6	42.1 (37.3;47.2)	0.2;157.4	57.0 (48.5;63.2)	0.5;142.0
Distance to the colony (km)	63.7 (50.3;78.0)	0.7;154.8	88.6 (71.9;105.7)	0.3;278.1	96.6 (81.8;110.5)	18.3;270.6
Bathymetry (m)	−238.0 (−284.0;−190.4)	−1480.9;−6.0	−242.5 (−265.5;−220.8)	−1465.6;−2.6	−358.7 (−397.9;−320.3)	−2253.9;−8.4
Seal occurrence index	6.6 (5.9;7.4)	1;10	6.4 (6.0;6.9)	1;10	4.8 (4.3;5.4)	1;10
Log(purse-seiner catch (tonnes)+1)	2.9 (1.4;4.4)	0.0;9.9	3.2 (2.3;4.2)	0.0:10.2	1.8 (1.0;2.6)	0.0;9.4
Log(trawler catch (tonnes)+1)	2.9 (1.9;3.8)	0.0;6.6	2.6 (2.4;2.9)	0.0;6.6	3.6 (3.2;4.0)	0.0;6.8
Sea surface temperature (°C)	16.6 (16.4;16.8)	13;19	16.6 (16.4;16.8)	13;19	16.9 (16.6;17.1)	13;19

We considered gannet habitat use during the daytime foraging and nighttime resting periods and in relation to their nighttime resting behavior (rest at colony or rest at sea) during October–November between 2008 and 2015, estimated with mixed linear models accounting for repeated measures within individual and year.

Testing prediction 2: During the day, gannets ventured into areas with higher risk of seal encounter compared to nighttime (Fig. 4a, Table 1), but still selected foraging areas that reduced seal co-occurrence (Fig. 2, Supplementary Note 3). Gannets competed with fisheries during the day, and were more likely to forage in areas with an increase in average catch of small pelagic fish by purse-seiners, relative to nighttime (Fig. 4c, Supplementary Note 4, Supplementary Table 5). Conversely, gannets were less likely to forage during the day, than rest at night, in areas with high average catch of hake by trawlers (Fig. 4d, Supplementary Table 5). On average, daytime gannet foraging areas had higher average catch of small pelagic fish by purse-seiners and lower average catch of hake by trawlers, compared to gannet nighttime resting areas (Table 1). However, at a daytime scale, foraging habitat selection of gannets did not rely on fisheries (Fig. 2, Supplementary Note 3).

## Discussion

Using a multi-year, large electronic tracking data set of gannet and seal at-sea movements and fisheries’ log-books, we validated our hypothesis: both fear and competition contribute to shaping regional gannet spatial ecology in the Benguela upwelling ecosystem. Gannets foraged in risky and competitive areas during the day. They also used movement tactics aiming to reduce spatial overlap with seals, especially during the riskiest night period. This involved coming back to the colony or performing a marked offshore spatial shift to safer waters. Our study therefore adds substantial support to the idea that seascape ecology must account for the fear factor^3,6,11^.

Interestingly, studies of zooplankton vertical migration first pointed to the LoF, but this concept has been mainly studied within terrestrial landscapes^4,5^. Indeed, so far only 4.3% (10/230, ISI Web of Science April 2021) of publications which addressed the LoF^2,5^ did so in a marine context. Most such studies focused on coastal reef habitats^12,13^, to the neglect of pelagic ecosystems^3,14^ and large spatial scales^11,15^. We fill these major gaps, by providing, to the best of our knowledge, the first evidence for a SoF in an upwelling ecosystem, at a regional scale. Our analyses confirm that seabirds avoid seals^16^ whenever they can, but in the Benguela upwelling ecosystem other top predators add layers of complexity to the pelagic fearscape. Notably, great white sharks (*Carcharodon carcharias*) are known to target both seals and seabirds^17^, even though they are far less abundant than seals. Orcas (*Orcinus orca*) may also take seals and great white sharks. We are therefore calling for SoF studies considering trophic networks within pelagic ecosystems, to rigorously understand fear effects on the spatiotemporal distribution of aquatic animals^6^, and their population consequences^5^.

The Benguela ecosystem is being challenged by the combined effects of climate change and overfishing, threatening a series of endemic seabird species: Cape gannet, Cape and Bank Cormorants (*Phalacrocorax capensis* and *P. neglectus*), and African penguin (*Spheniscus demersus*). In this context, marine protected areas (MPAs) are an effective way to safeguard the integrity of ecosystems, and of threatened seabirds^18^. However, MPAs have so far been designed and implemented on the basis of knowledge of seabird critical foraging habitats, to the neglect of the SoF imposed onto seabirds by their potential predators. Considering our fearscape study in the Benguela upwelling ecosystem, we posit that seascape ecology and forthcoming marine management should consider the SoF when designing MPAs.

## Methods

### Cape gannet movement tracking

The study took place in the Western Cape, South Africa, where we studied chick-rearing Cape gannets from Malgas Island (33.05° S, 17.93° E) during October–November from 2008 to 2015 (Fig. 1). We caught birds using a pole fitted with a loop and fitted 197 adult Cape gannets (22 in 2008, 16 in 2009, 38 in 2010, 11 in 2011, 29 in 2012, 29 in 2013, 23 in 2014, and 29 in 2015) with GPS-loggers (2008: GPS mass 65 g, i.e., 2.4 % adult body mass, Technosmart, Rom. 2009–2010: GPS mass 45 g, i.e., 1.7% adult body mass, Technosmart, Rom. From 2011: GPS mass 30 g, 1.1% of bird body mass, Catnip Technologies, Hong-Kong). Loggers were attached to the lower back with waterproof Tesa® tape and recorded position at a regular 30-s to 2-min intervals, reinterpolated over 1-min intervals. Devices were recovered after one foraging trip lasting a few hours to one week. Bird handling and tracking using these procedures do not have a measurable impact on foraging behavior^19,20^. We caught adult birds at-random from the colony, and previous studies showed that this resulted in a well-balanced sex-ratio preventing confounding sex effects^21^. All experiments were performed under permit from South African National Parks with respect to animal ethics (N° RYAP/AGR/001-2002/V1).

### Cape gannet movement tactics and behavioral phases

We identified two movement trip tactics for Cape gannets: After their daytime foraging activities, some birds returned to the colony at night (*rest at colony* tactic) while others spent all the night at sea (*rest at sea* tactic). Within the GPS tracks of gannets from these two categories, we discriminated resting, foraging, and commuting phases, with a segmentation-clustering method based on smoothed speed (i.e., speed smoothed over two steps before and after the focal location) and turning angle measured at constant step length. This corresponded to the angle between the focal location, the first location entering a circle of radius equal to the median step length, and the last location inside the circle^22^. We fitted behavioral identification with the *segclust2d* package^23^ for the R software^24^. See complete details on behavioral classification for Cape gannets tracks in Appendix 1 in Courbin et al.^25^.

### Cape fur seal movement tracking and the seascape of fear

We assessed the at-sea spatial distribution of Cape fur seals, a predator of Cape gannet fledglings^7^ and adults (Supplementary Data 1). We used Argos data collected from 25 lactating female seals before (2003 and 2004) and again concomitantly with gannet tracking (2012 and 2014). Seals were tracked during the same period of the year as gannets (i.e., September to November). Adult females nursing pups were selected at random and captured using a modified hoop net. Once restrained, anesthesia was induced using isoflurane gas delivered via a portable vaporizer (Stinger, Advanced Anesthesia Specialists, Gladesville, New South Wales, Australia). A satellite tag was glued to the guard hairs on the upper back. Individuals were allowed to recover from the anesthesia and resumed normal behavior within 45 min of capture. Throughout the process, the animals’ breathing was closely monitored and their flippers were repeatedly flushed with seawater to prevent hyperthermia. Seals were equipped with Argos satellite transmitters at three colonies (Fig. 1): Kleinsee (29°35’09”S, 16°59’56”E) located ~400 km to the North of the gannet colony (*n* = 8 seals in 2003 and 2004); Vondeling Island (33°09’11”S, 17°58’57”E), ~12 km away from the gannet colony (*n* = 12 seals in 2012 and 2014); and Geyser Rock (34°41’19”S, 19°24’49”E) located ~230 km to the South of the gannet colony (*n* = 5 seals in 2003). Seals at Vondeling Island were equipped with Argos-linked Spot-6 position transmitting tags (Wildlife Computers) following deployment procedures outlined in Kirkman et al.^26^. Seals at Kleinsee and Geyser Rock were equipped with ST18 and ST20 satellite-linked platform terminal transmitters (Telonics, Mesa, USA), as detailed in Skern-Mauritzen et al.^27^. Devices collected a well-balanced number of Argos locations during the day (*n* = 6080 locations) and at night (*n* = 6501 locations). See full details on seal tracking in Supplementary Table 6. All fieldwork was permitted by the Animal Ethics Committee of the Department of Environmental Affairs and Tourism’s Marine and Coastal Management branch, which at the time was the management authority of South Africa’s marine and coastal environment (Ref: DEAT2006-06-23).

We modeled both daytime and nighttime at-sea occurrences of seals for each colony with resource selection functions (RSF)^28,29^, a proxy of the fear effect for Cape gannets. RSF compared environmental features of seal’s at-sea Argos positions (i.e., further 500 m than the colony) with five times more random locations that captured the breadth of environmental conditions available to seals. We sampled random locations for each individual within the yearly area used by seals from each colony, delineated by the 95% kernel utilization distribution of the Argos locations of all seals of the colony. RSF were fitted with a generalized linear mixed model with a binomial distribution for errors. As environmental variables, we considered bathymetry (m), the slope of the bathymetry (°) and the distance to the colony (km) within the RSF. These variables were not highly correlated (|*r*| ≤ 0.61) and had low collinearity with a variance inflation factor VIF < 2^30^. All continuous predictors were centered and scaled. Following statistical recommendations in Muff et al.^29^, we added random intercept for seal ID with a large fixed variance, a random slope for each predictor and weighted random locations by 1000. We assessed the robustness of the RSF using a leave-one-out cross validation with iteratively one of the individuals representing the testing set and the other seals representing the training set^31,32^. RSF with a high predictive power had a high average Spearman’s rank correlation (${\bar{r}}_{s}$) between the rank of the RSF scores (relative probabilities of seal occurrence) split into ten bins and the area-adjusted frequency of the Argos locations^31^. We ran RSF with the *glmmTMB* package^33^ for the R software^24^.

Finally, we spatially predicted the binned RSF scores (10 bins) for each seal colony within our study area^34^. We assessed the seascape of fear by overlapping the three colony-specific RSF maps and retaining the maximum value among the three maps for each pixel.

### Co-occurrence of Cape gannets and fisheries

To assess the influence of fisheries on gannet movements we used vessel log-book records between 2008 and 2015. We mapped the yearly catch distribution of trawlers targeting hake (*Merluccius capensis*) with a 20 × 20 nautical mile resolution grid and of purse-seiners targeting anchovy (*Engraulis capensis*) and sardine (*Sardina pilchardus*) with a 10 × 10 nautical mile resolution grid. These data were made available by the branch: Fisheries Management of the Department of Forestry, Fisheries and the Environment of the Republic of South Africa.

### Daytime gannet foraging habitat selection

We assessed whether Cape gannets experienced different risks of encountering seals and different levels of competition with fisheries, according to their movement tactics. For this analysis, we only kept daytime foraging locations, and estimated foraging habitat selection of gannets with a RSF for each tactic^28,29^. Thereby, we fitted a generalized linear mixed model with a binomial distribution for errors with random intercept for gannet ID (with a large fixed variance) and year, random slope for each predictor and weighted available locations by 1000. We determined availability using a design adapted for central place foragers in habitat selection studies, considering that individuals used areas close to the colony more frequently than elsewhere^35,36^. For each observed foraging trip, we simulated 10 tracks that started at the same location as the observed trip (i.e., at the colony) using a first-order vector autoregressive model^37^. Simulated tracks considered no habitat preference, while respecting constraints on trip structure (duration and travel speed)^37^. For each simulated track, we then determined available foraging locations using the segmentation-clustering method described above (see “Cape gannet movement tactics and behavioral phases” section). RSF included daytime seal encounter risk, as well as the presence of purse-seiners and trawlers, assessed through the spatial distribution of their yearly catches (log(tonnes + 1)). All continuous predictors were centered and scaled, and had low collinearity (|*r*| ≤ 0.62, VIF < 2)^30^. We assessed RSF robustness as previously described, but using a *k*-fold cross validation with iteratively 80% of the individuals representing the training set and the 20% remaining birds representing the testing set^31,32^. We ran RSF with the *glmmTMB* package^33^ for the R software^24^.

### Cape gannet diel at-sea habitat use

We tested whether Cape gannets adjusted their at-sea behavior according to the presence of seals and fishing boats between relatively safe daytime and risky nighttime. For this purpose, we only considered gannets that did not return to the colony at night (*n* = 142 individuals, 72% of our sample). We estimated the probability of daytime foraging and nighttime resting, depending on both fishing activities and seal encounter risk. Thereby, we calculated daytime seal encounter risk for daytime foraging locations and nighttime seal encounter risk for nighttime resting locations. We used a generalized linear mixed model with a binomial distribution for errors and individual ID and year as random intercepts. Models also included the average monthly sea surface temperature (SST, °C) to test whether by moving away from risky areas to rest, gannets may also benefit from better thermoregulation conditions, i.e., higher SST. SST data were extracted from Aqua MODIS satellite imagery, with a 4-km-resolution grid (NASA Goddard Space Flight Center, Ocean Ecology Laboratory, Ocean Biology Processing Group; (2018): Aqua MODIS Sensor Ocean Color Data, NASA OB.DAAC.). All continuous predictors were centered and scaled. We tested candidate models with linear or nonlinear effects (natural spline with df = 4) for predictors, and selected the best model using the Akaike’s Information Criterion corrected for finite sample size. All candidate models did not include highly correlated variables (|*r*| < 0.4) and had low collinearity with VIF < 2^30^. We fitted models with the *lme4* package^38^ and model selection with the *MuMIn* package^39^ for the R software^24^.

### Reporting summary

Further information on research design is available in the Nature Research Reporting Summary linked to this article.

## Supplementary information

Supplementary Information

Description of Additional Supplementary Files

Supplementary Data 1

Reporting Summary

## Data Availability

Data are openly available in figshare at 10.6084/m9.figshare.17299094^40^, except Argos data for Cape fur seals that are under embargo until the end of 2023 as they are being used in the PhD thesis of Mduduzi Seakamela at the Department of Forestry, Fisheries and the Environment, Branch Ocean and Coasts, Cape Town, South Africa.

## References

[1] Pittman, S. J. (ed.). *Seascape Ecology* (John Wiley & Sons, 2017).

[2] Laundré JW, Hernández L, Altendorf KB (2001). Wolves, elk, and bison: reestablishing the ‘landscape of fear’ in Yellowstone National Park, USA. Can. J. Zool..

[3] Wirsing AJ, Heithaus MR, Frid A, Dill LM (2008). Seascapes of fear: evaluating sublethal predator effects experienced and generated by marine mammals. Mar. Mammal. Sci..

[4] Courbin N (2018). Zebra diel migrations reduce encounter risk with lions at night. J. Anim. Ecol..

[5] Gaynor KM, Brown JS, Middleton AD, Power ME, Brashares JS (2019). Landscapes of fear: spatial patterns of risk perception and response. Trends Ecol. Evolution.

[6] Ainley DG, Ballard G (2012). Non-consumptive factors affecting foraging patterns in Antarctic penguins: a review and synthesis. Polar Biol..

[7] Makhado AB, Crawford RJ, Underhill LG (2006). Impact of predation by Cape fur seals *Arctocephalus pusillus pusillus* on Cape gannets *Morus capensis* at Malgas Island, Western Cape, South Africa. Afr. J. Mar. Sci..

[8] Crawford RJM (2007). Trends in numbers of Cape gannets (*Morus capensis*) 1956/1957 – 2005/2006, with a consideration of the influence of food and other factors. ICES J. Mar. Sci..

[9] Cohen LA (2014). Changes in prey availability impact the foraging behaviour and fitness of Cape gannets over a decade. Mar. Ecol. Prog. Ser..

[10] Ropert-Coudert Y (2004). A fine-scale time budget of Cape gannets provides insights into the foraging strategies of coastal seabirds. Anim. Behav..

[11] Matthews CJD, Breed GA, LeBlanc B, Ferguson SH (2020). Killer whale presence drives bowhead whale selection for sea ice in Arctic seascapes of fear. Proc. Natl Acad. Sci. USA.

[12] Heithaus MR (2007). State-dependent risk-taking by green sea turtles mediates top-down effects of tiger shark intimidation in a marine ecosystem. J. Anim. Ecol..

[13] Catano LB (2016). Reefscapes of fear: predation risk and reef heterogeneity interact to shape herbivore foraging behaviour. J. Anim. Ecol..

[14] Bishop AM, Brown CL, Sattler R, Horning M (2020). An integrative method for characterizing marine habitat features associated with predation: a case study on juvenile steller sea lions (*Eumetopias jubatus*). Front. Mar. Sci..

[15] Hammerschlag N (2015). Evaluating the landscape of fear between apex predatory sharks and mobile sea turtles across a large dynamic seascape. Ecology.

[16] Masello JF, Kato A, Sommerfeld J, Mattern T, Quillfeldt P (2017). How animals distribute themselves in space: Variable energy landscapes. Front. Zool..

[17] Wcisel M, O’Riain MJ, de Vos A, Chivell W (2015). The role of refugia in reducing predation risk for Cape fur seals by white sharks. Behav. Ecol. Sociobiol..

[18] Pichegru L, Grémillet D, Crawford RJM, Ryan PG (2010). Marine no-take zone rapidly benefits endangered penguin. Biol. Lett..

[19] Grémillet D (2004). Offshore diplomacy, or how seabirds mitigate intra-specific competition. Mar. Ecol. Prog. Ser..

[20] Pichegru L (2007). Foraging behaviour and energetics of Cape gannets *Morus capensis* feeding on live prey and fishery discards in the Benguela upwelling system. Mar. Ecol. Prog. Ser..

[21] Lewis S (2002). Sex-specific foraging behaviour in a monomorphic seabird. Proc. R. Soc. Lond.: B Biol. Sci..

[22] Patin R, Etienne M-P, Lebarbier E, Chamaillé-Jammes S, Benhamou S (2020). Identifying stationary phases in multivariate time series for highlighting behavioural modes and home range settlements. J. Anim. Ecol..

[23] Patin, R., Etienne, M.-P., Lebarbier, E. & Benhamou, S. Segclust2d: Bivariate segmentation/clustering methods and tools. R package version 0.1.0. https://cran.r-project.org/web/packages/segclust2d (2018).

[24] R Development Core Team. *R: A language and environment for statistical computing*. Version 3.6.2. http://www.R-project.org/ (R Foundation for Statistical Computing, 2019).

[25] Courbin N (2020). The dance of the Cape gannet may contain social information on foraging behaviour. Anim. Behav..

[26] Kirkman SP (2019). Dive behaviour and foraging effort of female Cape fur seals *Arctocephalus pusillus pusillus*. R. Soc. Open Sci..

[27] Skern-Mauritzen M (2009). Do inter-colony differences in Cape fur seal foraging behaviour reflect large-scale changes in the northern Benguela ecosystem?. Afr. J. Mar. Sci..

[28] Manly, B., McDonald, L., Thomas, D. L., McDonald, T. L. & Erickson, W. P. *Resource Selection by Animals: Statistical Design and Analysis for Field Studies* (Springer Science and Business Media, 2002).

[29] Muff S, Signer J, Fieberg J (2020). Accounting for individual-specific variation in habitat-selection studies: efficient estimation of mixed-effects models using Bayesian or frequentist computation. J. Anim. Ecol..

[30] Dormann CF (2013). Collinearity: a review of methods to deal with it and a simulation study evaluating their performance. Ecography.

[31] Boyce MS, Vernier PR, Nielsen SE, Schmiegelow FKA (2002). Evaluating resource selection functions. Ecol. Model..

[32] Roberts DR (2017). Cross-validation strategies for data with temporal, spatial, hierarchical, or phylogenetic structure. Ecography.

[33] Brooks ME (2017). glmmTMB balances speed and flexibility among packages for zeroinflated generalized linear mixed modeling. R. J..

[34] Morris LR, Proffitt KM, Blackburn JK (2016). Mapping resource selection functions in wildlife studies: Concerns and recommendations. Appl. Geogr..

[35] Monsarrat S (2013). How predictability of feeding patches affects home range and foraging habitat selection in avian social scavengers?. PLoS ONE.

[36] Courbin N (2018). Short-term prey field lability constrains individual specialisation in resource selection and foraging site fidelity in a marine predator. Ecol. Lett..

[37] Raymond B (2015). Important marine habitat shift off east Antarctica revealed by two decades of multi-species predator tracking. Ecography.

[38] Bates D, Maechler M, Bolker B, Walker S (2015). Fitting linear mixed-effects models using lme4. J. Stat. Softw..

[39] Barton, K. MuMIn: Multi-Model Inference. R package version 1.43.17 (2020).

[40] Courbin, N. et al. dataset/Seascapes_of_fear_and_competition_shape_regional_seabird_movement_ecology. *Figshare*. 10.6084/m9.figshare.17299094 (2021).

[41] Pante E, Simon-Bouhet B (2013). marmap: A package for importing, plotting and analyzing bathymetric and topographic data in R. PLoS ONE.

