# Supplementary material for: Seascapes of fear and competition shape regional seabird movement ecology
Source: Commun Biol. 2022 Mar 4;5:208. doi: 10.1038/s42003-022-03151-z (PMC8897475; doi:10.1038/s42003-022-03151-z)
Supplement: Supplementary file 1 — Supplementary Information [file 42003_2022_3151_MOESM1_ESM.pdf]

Supplementary Information for:

Seascapes of fear and competition shape regional seabird movement ecology

Nicolas Courbin*†, Lorien Pichegru, Mduduzi Seakamela, Azwianewi Makhado, Michael Meÿer, Pieter G. H. Kotze, Steven A. Mc Cue, Clara Péron & David Grémillet*†

*Corresponding author. Email: ncourbin@gmail.com; david.gremillet@cebc.cnrs.fr

† These authors contributed equally to this work

Supplementary Note 1 - ***The "seal-scape" of fear***

RSFs correctly predicted day/night seal distribution for each colony (Supplementary Table 1). The average Spearman rank correlation had moderate value ranging between 0.63 and 0.46, but areas with the highest predicted occurrence were also the most used by seals, as expected for robust models (*1*, *2*). Seals used similar space during day and night (Supplementary Figure 1). Overall, seals more likely occurred in areas with a depth varying between -200m and 0m and with steep bathymetry (Supplementary Table 1, Supplementary Figure 1). Seals from the Vondeling colony (the seal colony closest to the studied gannet colony) also highly selected areas within a 200-km radius from their colony across the study period (Supplementary Table 1, Supplementary Figure 1).

Supplementary Note 2 - ***Cape gannet movement tactics***

Foraging Cape gannets displayed two main tactics: (1) Some (28%) came back to the colony at night, and foraged within 64 km, towards the South-West. 2) Most (72%) foraged farther (89 km) and spent the night at sea. When at sea, Cape gannets spent most of their time commuting towards, or between foraging areas (46% and 38% for birds spending the night at the colony or at sea, respectively), and actively foraging (37% and 41% for birds spending the night at the colony or at sea, respectively) at day. Birds which stayed at sea overnight spent on average 76% of the night resting.

Supplementary Note 3 - ***Daytime gannet habitat selection***

RSFs correctly predicted daytime gannet distribution for individuals spending the night at the colony (average Spearman rank correlation, $\overline{r_s}$ = 0.78) or at sea ($\overline{r_s}$ = 0.72). Gannets had similar daytime foraging habitat selection between both tactics (i.e., spending the night at the colony or at sea, Supplementary Table 2). Moreover, we tested a complete model with an interaction term between tactics and the three predictors, and did not find significant effect of tactics on the predictors. Predation risk/competition was the main driver of gannet foraging habitat selection, with birds strongly selecting foraging areas decreasing the risk of seal encounter (Supplementary Table 2). Further, our results suggested that gannets spending the night at sea tended to select daytime foraging areas with higher catches by purse-seiners than available

(Supplementary Table 2). However, daytime foraging habitat selection of gannets did not rely on trawlers for each tactic (Supplementary Table 2).

Supplementary Note 4 - *Diel gannet habitat use*

For the 142 gannets that spent the night at sea, we observed a clear spatial shift between locations dedicated to daytime foraging and nighttime resting activities. Gannets were closer to shore during daytime foraging periods (mean = 43.2 km) than during consecutive nighttime resting periods (mean = 56.3 km), resulting in a mean significant nighttime offshore spatial shift of 13.1 km (Supplementary Table 3).

The nighttime movement away from daytime foraging areas and in an opposite direction from the shore revealed that gannets managed both the risk of encountering seals and their feeding opportunities between day and night. The best model included significant nonlinear effects for predictors, except for the average annual catch of purse-seiners due to a fitting model issue (ΔAICc > 6262 and $w_i = 1$, Supplementary Table 4). Gannets were more likely to forage at day than rest at night in areas with a high risk of encountering seals and high average annual catch of purse-seiners (Supplementary Table 5). In contrast, gannets rested in areas farther from the shore and associated with a lower seal encounter risk during the night. This behavior allowed gannets to decrease their average risk of encountering seals by ca. 25% at night. Importantly, the risk of encountering seals was the most important driver of the difference in gannet space use between day and night (Supplementary Table 5). Gannets used areas to forage at day and rest at night independently of the increase in average annual catch of trawlers until a threshold value of ca. 5.5, beyond which they were more likely to rest than forage (Supplementary Table 5). Sea surface temperature also shaped diel gannet behavior. Areas with a SST ≥ 17°C significantly increased the likelihood that gannets rested at night, whereas colder water did not influence the probability of daytime foraging over nighttime resting (Supplementary Table 5). Yet, the mean SST for daytime foraging locations and nighttime resting locations was <17°C and their difference was only 0.3°C.

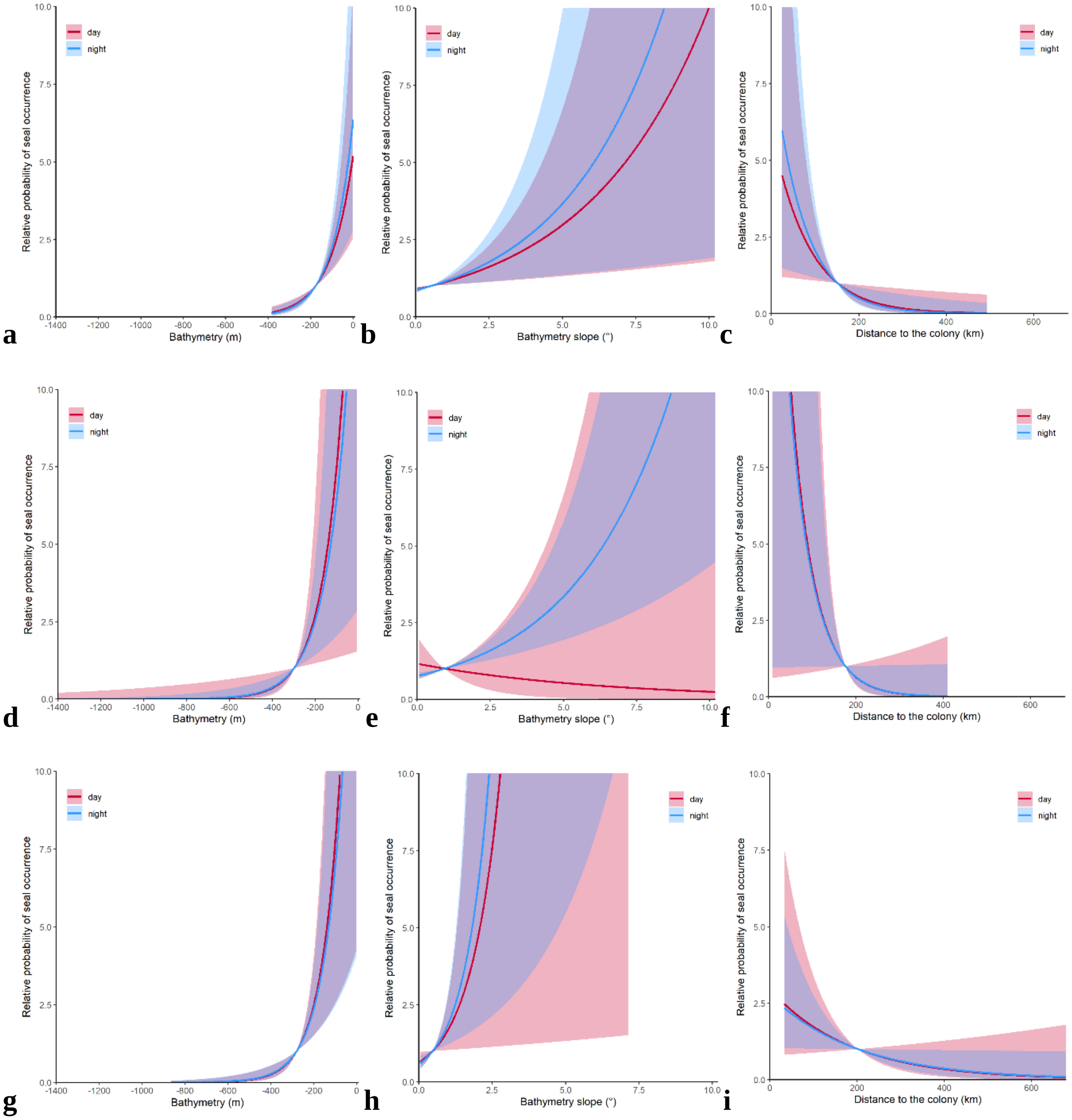

Supplementary Figure 1. Predicted relative probability of occurrence of seals for the (**a**, **b**, **c**) Vondeling colony, (**d**, **e**, **f**) Geyser Rock colony, and (**g**, **h**, **i**) Kleinsee colony in relation to (**a**, **d**, **g**) the bathymetry (m), (**b**, **e**, **h**) the slope of the bathymetry (°) and (**c**, **f**, **i**) the distance to the colony (km) for daytime and nighttime periods.

Supplementary Table 1. Relative probability of selection for Cape fur seals during the day and at night in 2003, 2004, 2012 and 2014, with the estimates (β), their 95% confidence interval (CI) and associated standard errors (SE). All models have the same random structure with random intercepts for individual IDs with a large fixed variance and random slopes for bathymetry, bathymetry slope and distance to the colony. Intercept was not shown and was 0 for all models as expected with our modelling approach.

Variable	Daytime		Nighttime	
	β (95% CI)	SE	β (95% CI)	SE
Vondeling colony, n = 12 seals				
Bathymetry (m)	1.29 (0.73;1.85)*	0.29	1.45 (0.80;2.10)*	0.33
Bathymetry_slope (°)	0.25 (0.06;0.43)*	0.09	0.29 (0.07;0.52)*	0.11
Distance to the colony (km)	-1.37 (-2.57;-0.16)*	0.61	-1.62 (-2.89;-0.36)*	0.65
k-fold: $\bar{r}_s$	0.63		0.61	
Geyser Rock colony, n = 5 seals				
Bathymetry (m)	4.62 (0.66;8.57)*	2.02	4.27 (1.63;6.91)*	1.35
Bathymetry_slope (°)	-0.25 (-1.23;0.74)	0.50	0.47 (0.26;0.68)*	0.11
Distance to the colony (km)	-2.07 (-4.46;0.33)	1.22	-2.01 (-4.05;0.03)	1.04
k-fold: $\bar{r}_s$	0.46		0.53	
Kleinsee colony, n = 8 seals				
Bathymetry (m)	3.35 (1.54;5.17)*	0.93	3.18 (1.48;4.88)*	0.87
Bathymetry_slope (°)	0.58 (0.04;1.13)*	0.28	0.70 (0.22;1.18)*	0.24
Distance to the colony (km)	-0.61 (-1.36;0.14)	0.38	-0.58 (-1.13;-0.02)*	0.28
k-fold: $\bar{r}_s$	0.47		0.51	

* means that 95% CI excludes 0.

Supplementary Table 2. Habitat selection of foraging behavior at daytime depending on the nighttime resting tactic for 197 Cape gannets from Malgas Islands during October-November between 2008 and 2015, with the estimates (β), their 95% confidence interval (CI) and associated standard errors (SE). Intercept was ca. 0 by definition and not shown.

Variable	β (95% CI)	SE
Tactic: rest at colony during the night, n = 53 birds, 8 years		
Seal occurrence index	-3.65 (-6.36;-0.95)*	1.38
Log(Purse-seiner catches+1)	-1.50 (-3.82;0.83)	1.19
Log(Trawler catches+1)	2.25 (-1.87;6.37)	2.10
Tactic: rest at sea during the night, n = 144 birds, 8 years		
Seal occurrence index	-3.52 (-4.85;-2.19)*	0.68
Log(Purse-seiner catches+1)	0.62 (-0.08;1.32)†	0.36
Log(Trawler catches+1)	-0.60 (-1.46;0.26)	0.44

* means that 95% CI excludes 0, † means that 90% CI excludes 0.

Notes: variance and covariance for the *rest at colony* model were fixed at 1000000 and 1000 for the gannet ID random intercepts, 1.00 and 1.00 for the year random intercepts, 98.5 and 9.9 for the random slopes for seal occurrence index, 59.2 and 7.7 for the random slopes for purse-seiner catches, and 207.6 and 14.4 for the random slopes for trawler catches. Variance and covariance for the *rest at sea* model were fixed at 1000000 and 1000 for the gannet ID random intercepts, 1.00 and 1.00 for the year random intercepts, 65.8 and 8.1 for the random slopes for seal occurrence index, 17.5 and 4.2 for the random slopes for purse-seiner catches, and 27.3 and 5.2 for the random slopes for trawler catches.

Supplementary Table 3. Linear mixed model estimating the difference in the distance to the shore between daytime foraging locations and nighttime resting locations for 142 Cape gannets during October-November between 2008 and 2015, with the estimates (β), their 95% confidence interval (CI) and associated standard errors (SE). We used random intercepts for individual ID and year.

Variable	β (95% CI)	SE
Intercept	56.27 (50.00;62.47)*	3.28
Period of the day (daytime)	-13.14 (-13.31;-12.95)*	0.09

* means that 95% CI excludes 0.

Notes: variance and covariance of the random intercepts were 464.5 and 21.55 for the gannet ID and 57.68 and 7.59 for the year.

Supplementary Table 4. Model selection results for candidate models estimating the probability of foraging over resting for 142 Cape gannets from Malgas Islands during October-November between 2008 and 2015, with the number of parameters (K), the log-likelihood (log-Lik), the Akaike's Information Criterion corrected for finite sample size (AICc), the difference in AICc values between individual models and the top model (ΔAICc), and the model weight (w_i). All candidate models had random intercepts for individual ID and year.

Model	K	log-Lik	AICc	ΔAICc	w_i
Spline (seal occurrence index, df=4) + purse-seiner + spline (trawler, df=4) + spline (SST, df=4)	16	- 66463.54	132959.1	0.00	1.00
Seal occurrence index + Purse-seiner + Trawler + SST	7	- 69603.59	139221.2	6262.09	0.00
Intercept only	3	- 82870.30	165746.6	32787.51	0.00
Spline (seal occurrence index, df=4) + spline (purse-seiner, df=4) + spline (trawler, df=4) + spline (SST, df=4)	19	*Fitting issue*			

Supplementary Table 5. Probability of foraging over resting for 142 Cape gannets from Malgas Islands during October-November between 2008 and 2015, with the estimates (β), their 95% confidence interval (CI) and associated standard error (SE).

Variable	β (95% CI)	SE
Intercept	-2.76 (-3.39;-2.11)	0.25
Seal occurrence index (spline 1)	2.73 (2.62;2.85)*	0.06
Seal occurrence index (spline 2)	5.78 (5.67;5.89)*	0.06
Seal occurrence index (spline 3)	6.78 (6.57;7.00)*	0.11
Seal occurrence index (spline 4)	1.93 (1.83;2.04)*	0.05
Purse-seiner	0.40 (0.38;0.42)*	0.01
Trawler (spline 1)	-0.24 (-0.33;-0.15)*	0.05
Trawler (spline 2)	0.26 (0.15;0.38)*	0.06
Trawler (spline 3)	-0.14 (-0.30;0.02)	0.08
Trawler (spline 4)	-0.79 (-0.92;-0.67)*	0.07
SST (spline 1)	-0.82 (-1.02;-0.62)*	0.09
SST (spline 2)	-1.21 (-1.40;-1.03)*	0.09
SST (spline 3)	-3.53 (-4.01;-3.05)*	0.22
SST (spline 4)	-3.18 (-3.46;-2.91)*	0.14

* means that 95% CI excludes 0.

Notes: variance and covariance of the random intercepts were 3.01 and 1.73 for the gannet ID (n = 142) and 0.40 and 0.63 for the year (n = 8).

Supplementary Table 6. Summary of satellite tracking of 25 female Cape fur seals in West of South Africa.

Colony	Monitoring period	Id	Number of Argos locations
Vondeling	Sept-Nov 2012	66310	718
Vondeling	Sept-Nov 2012	66311	515
Vondeling	Sept-Nov 2012	66350	564
Vondeling	Sept-Nov 2012	66354	605
Vondeling	Sept-Nov 2012	66393	589
Vondeling	Sept-Nov 2012	66394	630
Vondeling	Sept-Nov 2012	66407	588
Vondeling	Sept-Nov 2014	132189	500
Vondeling	Sept-Nov 2014	132190	344
Vondeling	Sept-Nov 2014	132191	419
Vondeling	Sept-Nov 2014	132192	402
Vondeling	Sept-Nov 2014	132193	448
Geyser Rock	Sept-Nov 2003	22716	879
Geyser Rock	Sept-Nov 2003	22753	432
Geyser Rock	Sept-Nov 2003	22772	860
Geyser Rock	Sept-Nov 2003	22780	617
Geyser Rock	Sept-Nov 2003	40844	418
Kleinsee	Sept-Nov 2003	14064	209
Kleinsee	Sept-Nov 2003	24190	662
Kleinsee	Sept-Nov 2003	40843	368
Kleinsee	Sept-Nov 2004	47711	69
Kleinsee	Sept-Nov 2004	47712	240
Kleinsee	Sept-Nov 2004	47714	392
Kleinsee	Sept-Nov 2004	47715	631
Kleinsee	Sept-Nov 2004	47717	482

Supplementary References

1. M. S. Boyce, P. R. Vernier, S. E. Nielsen, F. K. A. Schmiegelow, Evaluating resource selection functions. *Ecological Modelling* **157**(2-3), 281-300 (2002).
2. D. R. Roberts, V. Bahn, S. Ciuti, M. S. Boyce, J. Elith, G. Guillera-arroita, et al., Cross-validation strategies for data with temporal, spatial, hierarchical, or phylogenetic structure. *Ecography* **40**(8), 913-929 (2017).
