# Supplementary material for: Seascapes of fear and competition shape regional seabird movement ecology
Source: Commun Biol. 2022 Mar 4;5:208. doi: 10.1038/s42003-022-03151-z (PMC8897475; doi:10.1038/s42003-022-03151-z)
Supplement: Supplementary file 2 — Description of Additional Supplementary Files [file 42003_2022_3151_MOESM2_ESM.pdf]

Description of Additional Supplementary Files

File name: Supplementary Data 1
Description: Report of personal observations of Cape fur seals predating on adult Cape gannets.
