# Supplementary material for: Seascapes of fear and competition shape regional seabird movement ecology
Source: Commun Biol. 2022 Mar 4;5:208. doi: 10.1038/s42003-022-03151-z (PMC8897475; doi:10.1038/s42003-022-03151-z)
Supplement: Supplementary file 3 — Supplementary Data 1 [file 42003_2022_3151_MOESM3_ESM.docx]

Supplementary Data 1 - Report of personal observations of Cape fur seals predating on adult Cape gannets for:

Seascapes of fear and competition shape regional seabird movement ecology

Nicolas Courbin*†, Lorien Pichegru, Mduduzi Seakamela, Azwianewi Makhado, Michael Meÿer, Pieter G. H. Kotze, Steven A. Mc Cue, Clara Péron & David Grémillet*†

*Corresponding author. Email: ncourbin@gmail.com; david.gremillet@cebc.cnrs.fr

† These authors contributed equally to this work

**David Grémillet:** "I observed Cape fur seal predation events on adult Cape gannets offshore of Lambert’s Bay (Western Cape, South Africa) in January 2000, January 2002, December 2002, and December 2003. Each time, 5-10 adult Cape gannets resting at sea surface were attacked and killed by Cape fur seals."

**Azwianewi Makhado:** Cape fur seals have been observed predating on Cape gannets in October 2003 where 1 adult bird was observed at a distance ~150m. In January 2004 another adult cape gannet was observed being killed at sea. Furthermore, predation events were recorded on resting adult gannets between 1^st^ February to 28^th^ February and an average of 5 birds were predated offshore around Malgas island. Between March 2004 and October 2004 between 2 to 5 birds were also recorded killed by seals whilst resting at sea. In January 2005, further 5 birds were predated by seals offshore of Malgas island. Over the period Cape fur seals were recorded coming onshore at Lambert’s bay where a seal predated on adult Cape gannets breeding in 2005. This left the whole colony to be abandoned from breeding in that year.

**Michael Meyer:** Our field observations show that seals kill gannets at the sea surface, by dragging them below or by violently grabbing extremities and slamming the gannets from side to side against the water until sections of body flesh break off or in many cases with juvenile gannets, seals (especially small seals) bite the anal area and pull out the entrails from the anal area. In the past Department of Forestry, Fisheries and the Environment of the Republic of South Africa (DFFE) had annual research cruises to collect seal stomach contents and identify the diet at sea of seals. During sighting observations and grid searches we always saw 1-2 adult gannets dead at sea in which the breast or stomach had been taken by seals. This type of predation (seals) was very easy to identify by the damage to the carcass.

**Lorien Pichegru:** Seals killed about 20 gannets in the colony at Malgas Island [our study site] in 2005/2006. From 2017, seals were regularly accessing the colony early in the breeding season, during the incubating phase, killing a number of gannets in their way and generating large disturbance on the colony. From 2018, reports from the Southern African Foundation for the Conservation of Coastal Birds (SANCCOB) vet described lesions from seals on adult gannets (e.g. 45 in 2018, 6 in 2019). Later in the season, volunteers are posted on the island to chase potential predators, i.e. pelicans and seals. This volunteer program has been set in place by South African National Parks since 2005, thereby reducing predator’s impact on the colony but only from November onward.
