# Supplementary material for: Seascapes of fear and competition shape regional seabird movement ecology
Source: Commun Biol. 2022 Mar 4;5:208. doi: 10.1038/s42003-022-03151-z (PMC8897475; doi:10.1038/s42003-022-03151-z)
Supplement: Supplementary file 4 — Reporting Summary [file 42003_2022_3151_MOESM4_ESM.pdf]

nature research

Corresponding author(s): DBPR COMMSBIO-21-2557A

Last updated by author(s): Dec 16, 2021

Reporting Summary

Nature Research wishes to improve the reproducibility of the work that we publish. This form provides structure for consistency and transparency in reporting. For further information on Nature Research policies, see our Editorial Policies and the Editorial Policy Checklist.

Statistics

For all statistical analyses, confirm that the following items are present in the figure legend, table legend, main text, or Methods section.

n/a	Confirmed	
☐	☒	The exact sample size (*n*) for each experimental group/condition, given as a discrete number and unit of measurement
☐	☒	A statement on whether measurements were taken from distinct samples or whether the same sample was measured repeatedly
☐	☒	The statistical test(s) used AND whether they are one- or two-sided<br>*Only common tests should be described solely by name; describe more complex techniques in the Methods section.*
☐	☒	A description of all covariates tested
☐	☒	A description of any assumptions or corrections, such as tests of normality and adjustment for multiple comparisons
☐	☒	A full description of the statistical parameters including central tendency (e.g. means) or other basic estimates (e.g. regression coefficient) AND variation (e.g. standard deviation) or associated estimates of uncertainty (e.g. confidence intervals)
☐	☒	For null hypothesis testing, the test statistic (e.g. *F*, *t*, *r*) with confidence intervals, effect sizes, degrees of freedom and *P* value noted<br>*Give P values as exact values whenever suitable.*
☒	☐	For Bayesian analysis, information on the choice of priors and Markov chain Monte Carlo settings
☐	☒	For hierarchical and complex designs, identification of the appropriate level for tests and full reporting of outcomes
☒	☐	Estimates of effect sizes (e.g. Cohen's *d*, Pearson's *r*), indicating how they were calculated

Our web collection on statistics for biologists contains articles on many of the points above.

Software and code

Policy information about availability of computer code

Data collection	No software was used
Data analysis	R Development Core Team, version 3.6.2.

For manuscripts utilizing custom algorithms or software that are central to the research but not yet described in published literature, software must be made available to editors and reviewers. We strongly encourage code deposition in a community repository (e.g. GitHub). See the Nature Research guidelines for submitting code & software for further information.

Data

Policy information about availability of data

All manuscripts must include a data availability statement. This statement should provide the following information, where applicable:

- Accession codes, unique identifiers, or web links for publicly available datasets
- A list of figures that have associated raw data
- A description of any restrictions on data availability

Data are openly available in figshare at http://doi.org/10.6084/m9.figshare.17299094, except Argos data for Cape fur seals that are under embargo until end of 2023 as they are being used in the PhD thesis of Mduduzi Seakamela at the Department of Forestry, Fisheries and the Environment, Branch Ocean and Coasts, Cape Town, South Africa.

Field-specific reporting

Please select the one below that is the best fit for your research. If you are not sure, read the appropriate sections before making your selection.

☐ Life sciences ☐ Behavioural & social sciences ☒ Ecological, evolutionary & environmental sciences

For a reference copy of the document with all sections, see nature.com/documents/nr-reporting-summary-flat.pdf

Ecological, evolutionary & environmental sciences study design

All studies must disclose on these points even when the disclosure is negative.

Study description	Diel movement ecology of Cape gannets related to the seascape of fear and competition imposed by Cape fur seals and fisheries.
Research sample	197 adult Cape gannets from Malgas Island, South Africa<br>25 lactating female Cape fur seals at three colonies from South Africa
Sampling strategy	Animals were chosen randomly at their colonies. Telemetry studies have to balance the number of individuals tracked with the cost of electronic devices and potential individual/colony disturbance.
Data collection	Cape gannet data were collected with GPS loggers. Cape fur seal data were collected with Argos satellite transmitters. All authors gathered data.
Timing and spatial scale	Cape gannet data were collected each year during October-November from 2008 to 2015 with 11 to 38 individuals per year.<br>Cape fur seal were Argos tracked during September-November in 2003 (n=13), 2004 (n=8), 2012 (n=12) and 2014 (n=12).<br>We studied at-sea individual movements at a regional scale.
Data exclusions	No data were excluded
Reproducibility	Data were acquired by GPS/Argos devices. Field capture and acquisition program of loggers were precised in the manuscript.
Randomization	We caught adult birds at-random from the colony, and previous studies showed that this resulted in a well-balanced sex-ratio preventing confounding sex effects. Female seals were chosen randomly at their colonies.
Blinding	Data were acquired with GPS/Argos electronic devices.

Did the study involve field work? ☒ Yes ☐ No

Field work, collection and transport

Field conditions	Not relevant
Location	Cape gannet: South Africa, Malgas Island (33.05° S, 17.93° E)<br>Cape fur seal: South Africa, Kleinsee (29°35’09”S, 16°59’56”E) , Vondeling Island (33°09’11”S, 17°58’57”E), Geyser Rock (34°41’19”S, 19°24’49”E)
Access & import/export	Cape gannet: All experiments were performed under permit from South African National Parks with respect to animal ethics (N° RYAP/AGR/001—2002/V1).<br>Cap fur seal: All fieldwork was permitted by the Animal Ethics Committee of the then Department of Environmental Affairs and Tourism’s Marine and Coastal Management branch, which at the time was the management authority of South Africa’s marine and coastal environment (Ref: DEAT2006-06-23).
Disturbance	Cape gannet: loggers were attached to the lower back with waterproof Tesa® tape and recovered after one foraging trip lasting a few hours to one week. Bird handling and tracking using these procedures does not have a measurable impact on foraging behavior, based on previous studies.<br>Cape fur seal: Adult females nursing pups were selected at random and captured using a modified hoop net. Once restrained, anaesthesia was induced using isoflurane gas delivered via a portable vaporizer (Stinger, Advanced Anaesthesia Specialists, Gladesville, New South Wales, Australia). A satellite tag was glued to the guard hairs on the upper back. Individuals were allowed to recover from the anaesthesia and resumed normal behaviour within 45 min of capture. Throughout the process, the animals’ breathing was closely monitored and their flippers were repeatedly flushed with seawater to prevent hyperthermia.

Reporting for specific materials, systems and methods

We require information from authors about some types of materials, experimental systems and methods used in many studies. Here, indicate whether each material, system or method listed is relevant to your study. If you are not sure if a list item applies to your research, read the appropriate section before selecting a response.

Materials & experimental systems

n/a	Involved in the study
☒	☐ Antibodies
☒	☐ Eukaryotic cell lines
☒	☐ Palaeontology and archaeology
☐	☒ Animals and other organisms
☒	☐ Human research participants
☒	☐ Clinical data
☒	☐ Dual use research of concern

Methods

n/a	Involved in the study
☒	☐ ChIP-seq
☒	☐ Flow cytometry
☒	☐ MRI-based neuroimaging

Animals and other organisms

Policy information about studies involving animals; ARRIVE guidelines recommended for reporting animal research

Laboratory animals	The study did not involve laboratory animals.
Wild animals	Adult Cape gannets of both sexes and adult female Cape fur seals were caught, equiped and released at the capture site after manipulation.
Field-collected samples	The study did not involve samples collected from the field.
Ethics oversight	Cape gannet: All experiments were performed under permit from South African National Parks with respect to animal ethics (N° RYAP/AGR/001—2002/V1).<br>Cape fur seal: All fieldwork was permitted by the Animal Ethics Committee of the then Department of Environmental Affairs and Tourism's Marine and Coastal Management branch, which at the time was the management authority of South Africa's marine and coastal environment (Ref: DEAT2006-06-23).

Note that full information on the approval of the study protocol must also be provided in the manuscript.
